# The Functional Connectivity Landscape of the Human Brain

**DOI:** 10.1371/journal.pone.0111007

**Published:** 2014-10-28

**Authors:** Bratislav Mišić, Zainab Fatima, Mary K. Askren, Martin Buschkuehl, Nathan Churchill, Bernadine Cimprich, Patricia J. Deldin, Susanne Jaeggi, Misook Jung, Michele Korostil, Ethan Kross, Katherine M. Krpan, Scott Peltier, Patricia A. Reuter-Lorenz, Stephen C. Strother, John Jonides, Anthony R. McIntosh, Marc G. Berman

**Affiliations:** 1 Rotman Research Institute, Baycrest Centre, Toronto, Ontario, Canada; 2 Department of Radiology, University of Washington, Seattle, Washington, United States of America; 3 MIND Research Institute, Irvine, California, United States of America; 4 School of Nursing, University of Michigan, Ann Arbor, Michigan, United States of America; 5 Department of Psychology, University of Michigan, Ann Arbor, Michigan, United States of America; 6 Department of Psychology, Chungnam University, Daejeon, South Korea; 7 Department of Psychology, University of South Carolina, Columbia, South Carolina, United States of America; 8 Department of Psychology, University of Chicago, Chicago, Illinois, United States of America; Institute of Psychology, Chinese Academy of Sciences, China

## Abstract

Functional brain networks emerge and dissipate over a primarily static anatomical foundation. The dynamic basis of these networks is inter-regional communication involving local and distal regions. It is assumed that inter-regional distances play a pivotal role in modulating network dynamics. Using three different neuroimaging modalities, 6 datasets were evaluated to determine whether experimental manipulations asymmetrically affect functional relationships based on the distance between brain regions in human participants. Contrary to previous assumptions, here we show that short- and long-range connections are equally likely to strengthen or weaken in response to task demands. Additionally, connections between homotopic areas are the most stable and less likely to change compared to any other type of connection. Our results point to a functional connectivity landscape characterized by fluid transitions between local specialization and global integration. This ability to mediate functional properties irrespective of spatial distance may engender a diverse repertoire of cognitive processes when faced with a dynamic environment.

## Introduction

Functional brain networks are continually evolving on top of an anatomical skeleton to engender perception, thought and action. Patterns of functional connectivity may change either spontaneously [1]–[3], in response to task demands [4]–[7] or in response to disease or pharmacological interventions [8]–[10]. It is unclear, however, whether these various sources of connectivity change can be characterized in a principled way to predict brain states.

One hypothesis is that short-range functional connections are more stable than long-range connections. The laminar, cytoarchitectonic and columnar organization of the neocortex suggests that proximal areas may participate in similar functions [11]. As a result, short-range functional connections may be relatively stable and less likely to be affected by changes in task demands. Conversely, long-range functional connections between distal brain areas with dissimilar functions may be less stable and more likely to change due to task. Therefore, changes in tasks and psychological conditions may elicit relatively large changes in functional connectivity between distal areas and relatively small changes between proximal areas.

An alternative hypothesis is that task-dependent changes in functional connectivity do not depend on the distance between areas. Complex network theory suggests that, for optimal information processing, functional networks must simultaneously enable local segregation and global integration, thereby allowing for the interplay between specialization and integration of function [12]–[14]. To strike an adaptive balance between integration and segregation, task-dependent reconfiguration of functional networks would require flexible functional connectivity that is not biased by the distance between areas. According to this hypothesis, changes in brain states will be associated with equally stable short- and long-range functional connections.

Here we investigated whether the stability of a functional connection between two brain regions is affected by the length (distance spanned) of that connection. We define stability as a change in functional connectivity across two or more tasks or psychological conditions. We used the Euclidean distance between two regions as a proxy for the length of their functional connection. We analyzed data from four functional magnetic resonance imaging (fMRI) datasets, one magnetoencephalography (MEG) dataset and one positron emission tomography (PET) dataset. The data that we analyzed varied in task conditions, populations, preprocessing parameters, and acquisition parameters and yet even with all of this heterogeneity we demonstrate two key results across all studies. 1) Within a single condition, functional connectivity depends on spatial distance, i.e., short-range functional connections are stronger than long-range connections [a common finding [15]]. 2) Critically, the spatial distance between two regions does not affect the stability of their functional connectivity. Between conditions, changes in functional connectivity do not depend on connection length. Thus, both short- and long-range functional connections are equally likely to change and are equally stable.

While distance is not predictive of stability, whether a connection is between homotopic areas does predict stability. Namely, connections between homotopic areas do not change and therefore are the most stable connections. Importantly, we draw these conclusions based on data from a wide-range of tasks, populations, scanning environments, parcellation schemes, and preprocessing parameters indicating that our results are robust to these idiosyncrasies. These results suggest a simple yet fundamental characteristic of brain network dynamics; that changes in task result in alterations to the entire landscape of functional connections, independent of their lengths.

## Results

### Dataset Summaries and Rationale for Inclusion

Here we summarize the datasets that we used for our analyses. The interested reader can find all of the details for each individual study under the specific dataset sub-headings in the Materials and Methods section. However, these details are not necessary to understand our results and analyses. This section, and the next section, “Partial Least-Squares (PLS) Analysis Parameters,” are the only methods’ sections that are required to understand the results and analyses.

Methodological details regarding participants, tasks, and processing parameters for the six datasets used in this analysis are shown in Table 1. Two fMRI datasets contained data from clinically depressed patients and breast cancer patients to examine if *our results were robust for different clinical populations*. The third and fourth fMRI datasets involved healthy adults performing a learning task where participants learned to associate pseudowords to pictures, and a crossmodal (auditory-visual) attentional cuing task to examine if *our results were robust for on-task performance vs. resting-states*. The MEG dataset was also acquired using healthy adults participating in a visual learning paradigm. The PET data were derived from a study on memory recall in young and old adults. The MEG and PET datasets were included to examine if *our results were robust for different imaging modalities*. In the paragraphs below we describe these studies.

**Table 1:** Details for the neuroimaging studies that were analyzed.

Study	Modality	Groups	Conditions	ROIs	ParcellationScheme
**Depression**	fMRI	Depressed(N = 16) HealthyControl (N = 17)	4: Resting 1,Resting 2,Induced Rumination,Resting 3	116	AAL
**Breast Cancer**	fMRI	Chemotherapy(N = 16) Radiationtherapy (N = 16)Healthy Control(N = 16)	2: Pre-treatment,Post-treatment	116	AAL
**Learning**	fMRI	Healthy Control(N = 12)	6: Learningconditions	152	AAL/MNI hybrid
**Crossmodal**	fMRI	Healthy Control(N = 16)	4: Autitory-Visual Cue targetcombinations	229 voxels	Every 100th gray matter voxel
**MEG**	MEG	Healthy Control(N = 14)	3: LearningConditions	32 sources	N/A
**PET**	PET	Healthy Control(N = 12)	2: Memoryencoding andRecall	13 ROIs	N/A

Details for the PET study can be found in [31].

In the depression dataset [16], healthy individuals and individuals diagnosed with major depression had their resting-state connectivity assessed and this resting-state connectivity was compared to a condition where participants were induced to ruminate about two negative, self-generated, autobiographical memories. There was also a resting-state scan after this rumination phase to examine recovery after induced rumination. ***Here changes in connectivity were assessed for changes between rest and induced rumination***.

In the breast cancer dataset, women with breast cancer and healthy-aged matched controls had their brain connectivity assessed during resting-state epochs. Of the patients with breast cancer, half received chemotherapy treatment for breast cancer and half received radiation therapy for breast cancer. Connectivity was assessed before and after chemotherapy treatment, radiation therapy and an equivalent period of elapsed time for the healthy aged-matched controls. ***Here connectivity changes were due to treatment (i.e., chemotherapy and radiation therapy) vs. the mere passage of time for age-matched controls*** *.*

In the cross-modal dataset [17] participants responded to visual and auditory targets. A trial was composed of a stimulus (S1), a delay, and a second stimulus (S2). The two main task types were designed by manipulating the content of S1 and S2. For the Cue 1st (C1) tasks, S1 was a cue that signaled a response compatibility rule to a subsequent lateralized target (S2). In Target 1st (C2) tasks, S1 was the lateralized target followed by the cue stimulus (S2). In a compatible trial, a cue instructed participants to press a button on the same side (left or right) as the lateralized target. In an incompatible trial, participant’s pressed the button on the opposite side of the lateralized target. Unlike in the depression and breast cancer datasets, ***here connectivity changes were assessed by examining connectivity changes for changes in the task conditions such as trials when one must respond to a visual target preceded by an auditory cue vs. when one must respond to an auditory target preceded by a visual cue***.

In the learning dataset participants heard an auditory presentation of a pseudoword. After the presentation of the pseudoword participants saw a picture presented for 1 second and were told to intuitively decide if the pairing of pseudoword and picture was correct. There were two conditions: “Learning” and “No-Learning.” The “Learning” condition involved a higher co-occurrence of “correct” arbitrary object-pseudoword pairings compared to “incorrect” pairings. Participants learned this novel vocabulary of 30 concrete nouns over the course of five consecutive training blocks. Over the course of the five training blocks the ratio of “correct” to “incorrect” pairings increased. The “No-Learning” condition was structured similarly, but used a parallel set of pseudoword-object pairings and lacked a learning principle because each pairing occurred only once. ***Here changes in connectivity were measured as learning progressed***.

In the MEG dataset [18] participants performed two tasks: an active learning task and a choice reaction time task with no learning component. In the learning task, participants linked 2 scene pairs with 2 color pairs (4 associations) through trial-and-error. The general structure of a trial consisted of scene presentation, a delay interval and a color pair, after which the participants had to record their responses. Participants received on-screen, written feedback (correct/wrong) about their response choices. In the control task, participants were instructed in advance about which colors were correct and scenes preceding these colors were shown randomly. ***Here changes in connectivity were assessed for these different task conditions and for a different imaging modality (i.e., MEG)***.

In the PET dataset participants encoded and recalled word pairs. Effective connectivity was assessed using Structural Equation Modeling (SEM). ***Here changes in connectivity were assessed for yet another imaging modality (i.e., PET), for differences in memory recall by age (i.e., aging effects) and for effective connectivity rather than functional connectivity***.

### Correlating Functional Connectivity and Euclidean Distance

**Figure 1:**
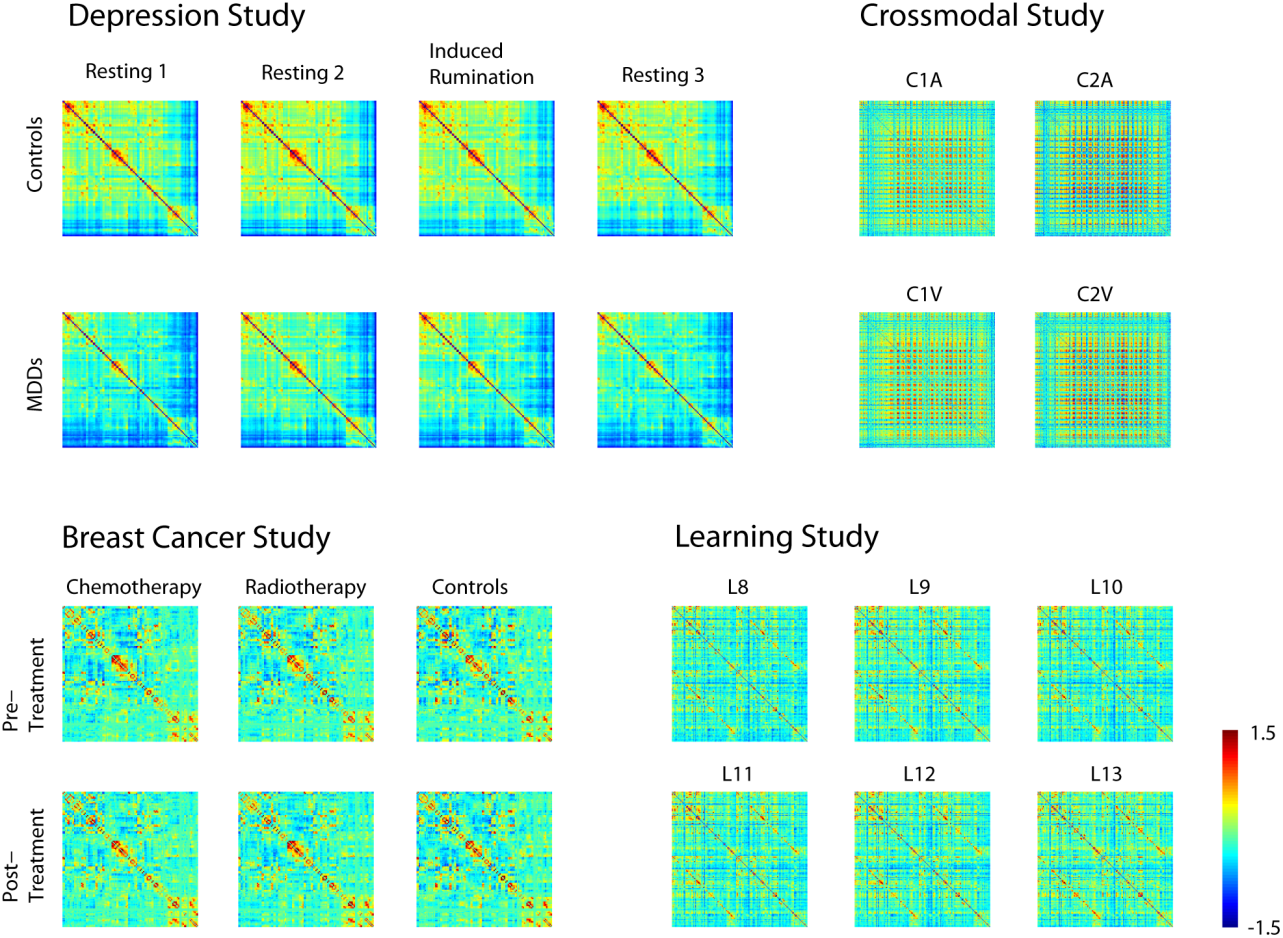
Fisher r-to-z-transformed correlation matrices for all studies exhibiting the correlation between all nodes used to analyze each study. For the PLS analyses, each participant’s matrix was used in the analysis.

The parcellation scheme used for each dataset is specified in Table 1. For datasets that were parceled into ROIs, time series were averaged across all voxels in each ROI. Functional connectivity matrices were then created by correlating the mean ROI timeseries with all other mean ROI timeseries separately for each individual participant and for each condition. Sample Fisher-transformed and group averaged functional connectivity matrices are shown separately for each condition (Fig. 1).

**Figure 2:**
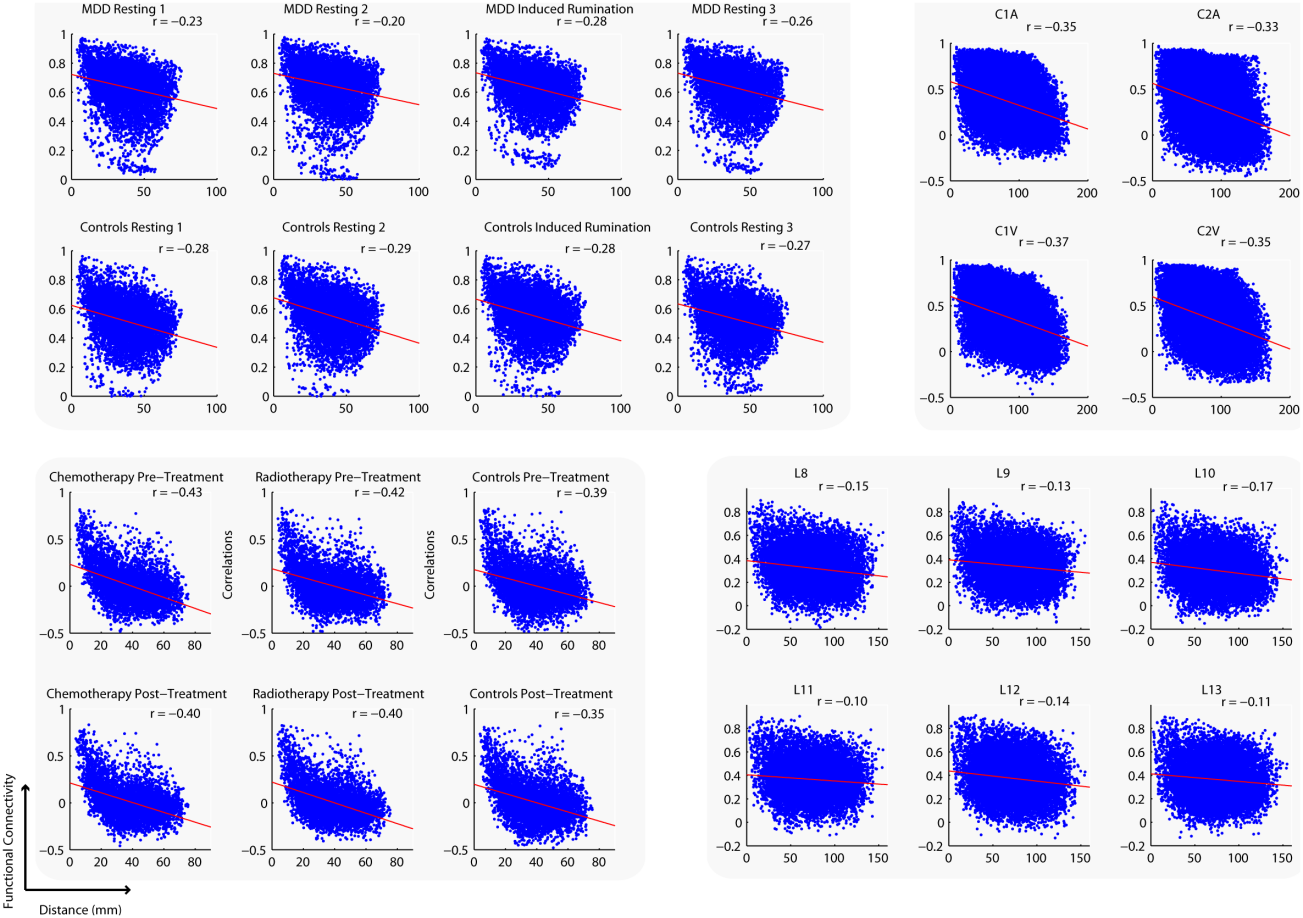
Relationship between the Euclidean distance of parcels and the correlation between parcel timeseries for each fMRI study and for each condition and participant group.

The Euclidean distance between ROI centroids was calculated and correlated with functional connectivity. Euclidean distance significantly predicted functional connectivity for each study, for each group and for each condition, such that ROIs that were spatially closer were more likely to be highly correlated (Fig. 2). This result has been found by other researchers [2], [15], [19] and may be expected given that contiguous brain areas participate in similar functions. In addition, studies find that contiguous brain areas share similar regional homogeneity [20], [21], i.e. temporal signatures, which may also indicate similar functionality. This aspect of functional neuroanatomy also results in the well-known spatial autocorrelation of the BOLD signal [22]. In summary, within a condition, Euclidean distance significantly predicts functional connectivity, i.e., connections between more proximal areas are more highly correlated.

### Correlating Changes in Functional Connectivity and Euclidean Distance

The results presented in the previous sub-section indicate that, within a condition, Euclidean distance and functional connectivity are negatively correlated, such that proximal areas tend to be more highly correlated with one another compared to more distal areas. In the present section, we show that there is no reliable relationship between Euclidean distance and changes in functional connectivity due to changes in task or group.

**Figure 3:**
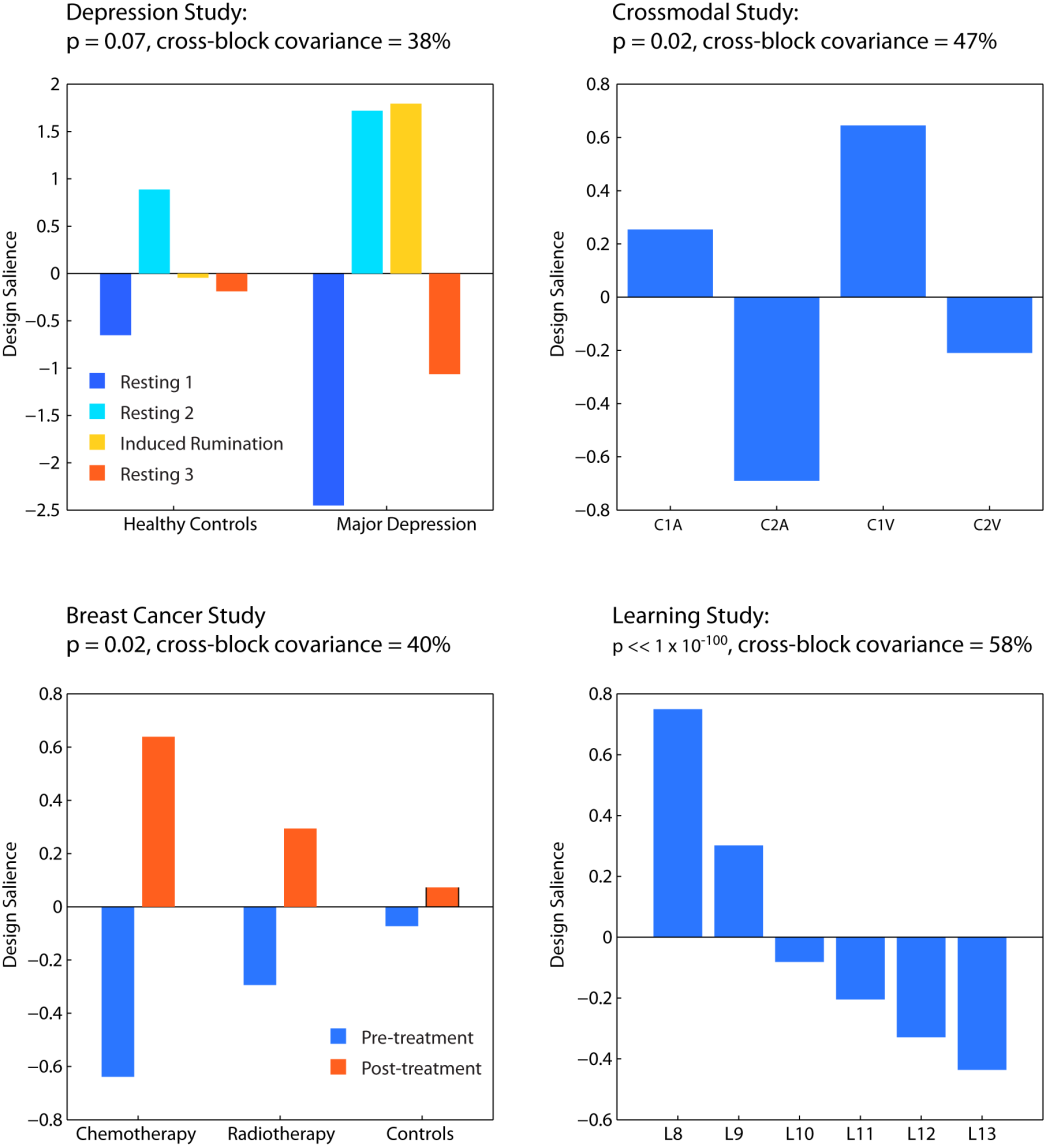
First Latent Variables (LVs) from each of the four fMRI Datasets as uncovered with PLS. The significance of the LVs and the amount of cross-block covariance explained are listed in the titles.

For each dataset we extracted participant- and condition-specific functional connectivity matrices and used a partial least-squares (PLS) analysis [23]–[25] to find connections that showed significant changes in functional connectivity across tasks and/or groups. The latent variables (LVs) from those analyses are shown in Fig. 3. Connection saliences are weights that describe the extent to which a connection expresses a latent variable or contrast, and therefore they express the degree to which a connection changes according to the latent variable pattern. For the depression dataset the greatest changes in functional connectivity occurred for the individuals diagnosed with depression who are more affected by the induced rumination condition compared to rest. These results are described in more depth in [16].

For the Breast Cancer dataset the greatest changes in functional connectivity occurred pre- and post-chemotherapy, followed by pre- and post-radiation therapy. The healthy group does not show reliable changes over an equivalent period of elapsed time (as expected given that they receive no intervention). Related breast cancer results are described in more depth in [26], [27].

In the Crossmodal dataset there is a significant interaction for changes in functional connectivity by cue position (1 vs. 2) and target type (visual vs. verbal). The Crossmodal results are described in more depth in [17]. Lastly, in the learning dataset there are significant changes in functional connectivity from the baseline reaction time condition with no learning (L8) compared to the other learning conditions as learning progresses. When comparing the greatest change in conditions, we compared the last learning session to the baseline reaction time task that required no learning.

The specific LV results are not critical for this investigation (please see the aforementioned studies for analyses and results specific to these studies). What is important is that for each study there were functional connections that significantly changed due to experimental manipulation (i.e. across groups and/or conditions). Based on these significant LVs, we can determine whether changes in functional connectivity are distance-dependent or distance-independent.

**Figure 4:**
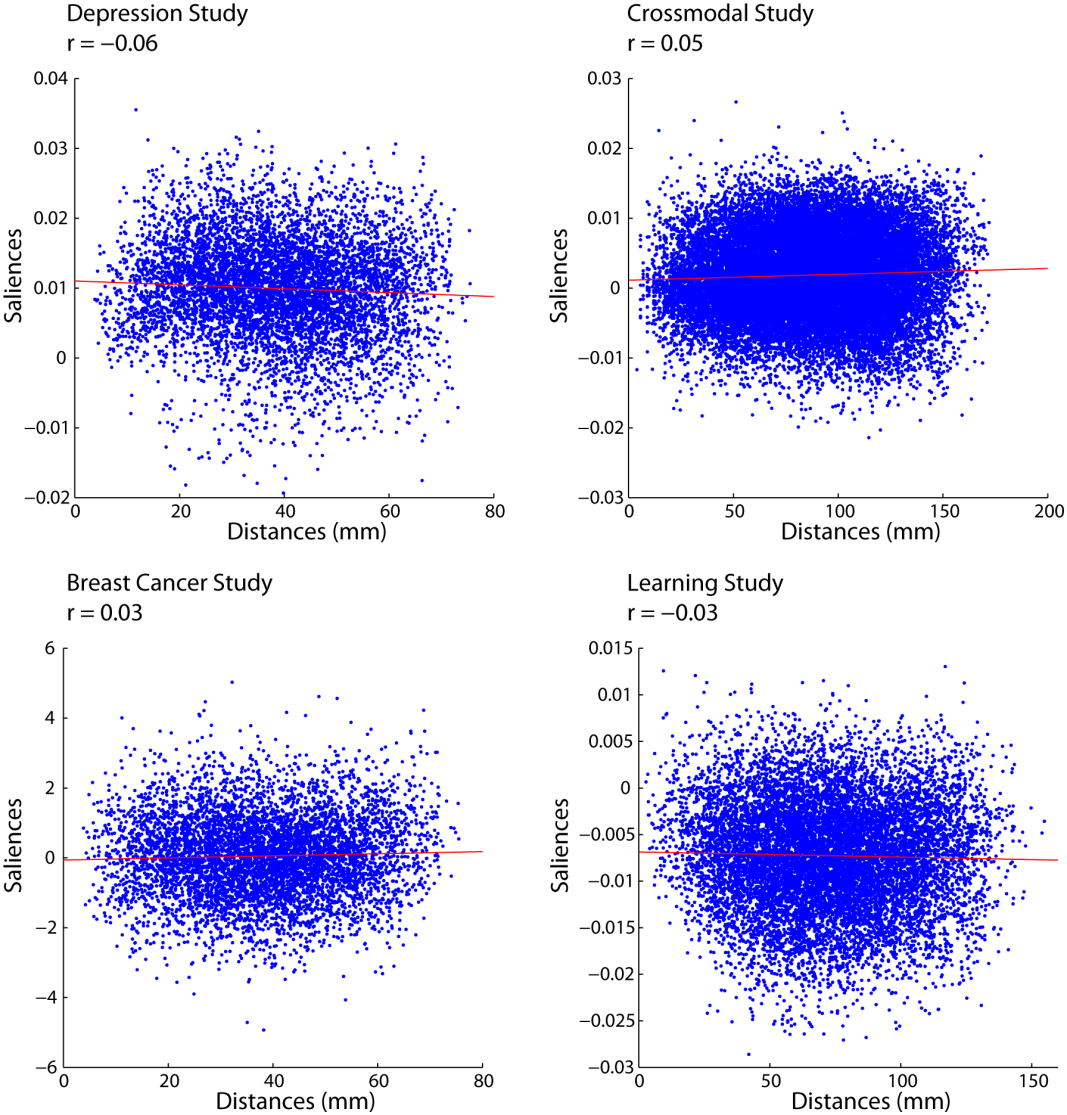
PLS Saliences for the first latent variable in each study plotted against Euclidean distance of each functional connection.

To determine whether the lengths of connections predicted connection changes we correlated the saliences of connection changes with Euclidean distance (Fig. 4). The result of that analysis showed no relationship between Euclidean distances and salience values (Fig. 4). These results suggest that all functional connections, independent of the distance between them, are equally likely to change.

**Figure 5:**
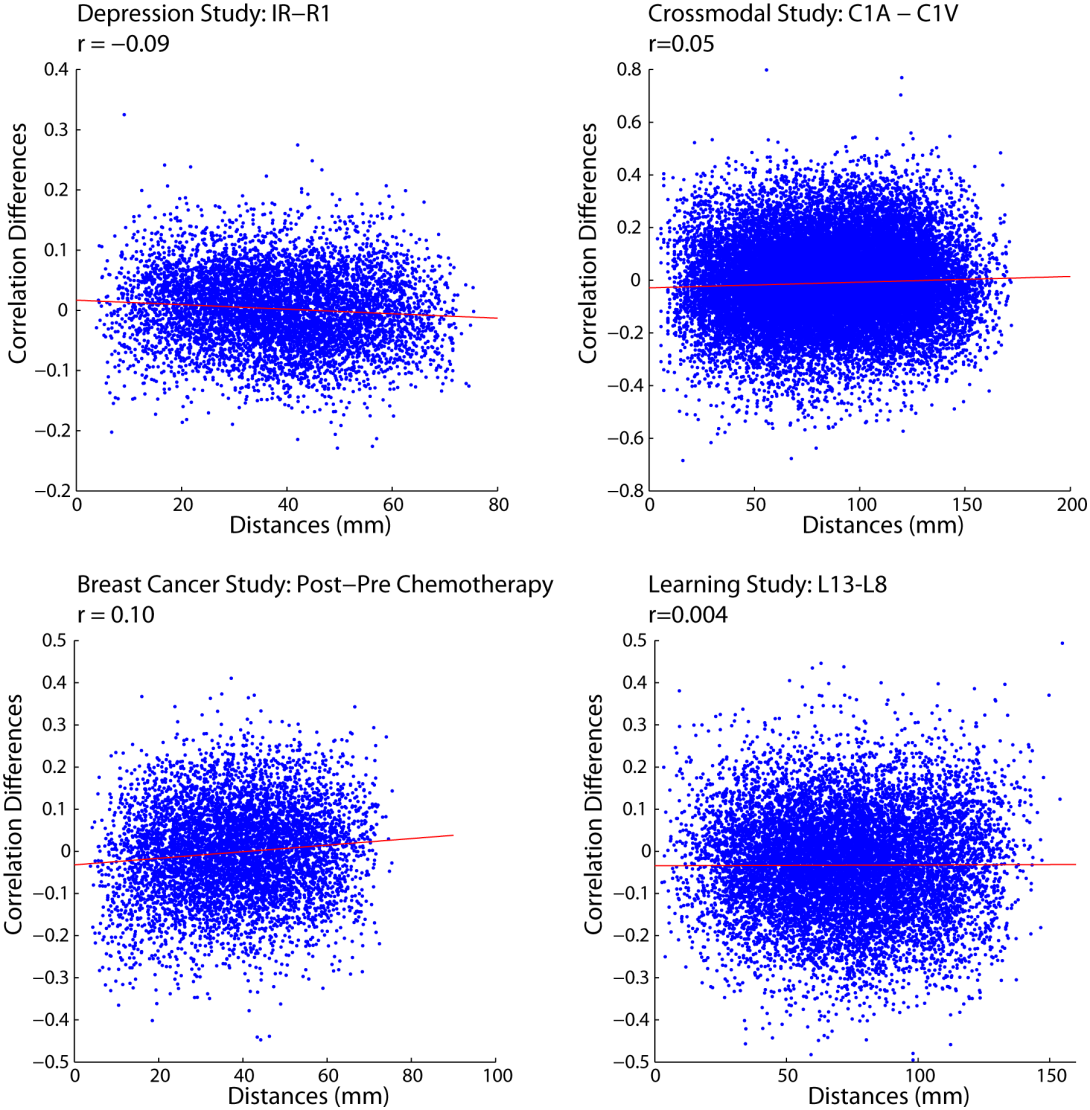
Changes in correlation between conditions of greatest difference in correlation plotted against Euclidean distance.

We then performed an analysis to examine how differences in correlation values between two conditions in the experiments might change with Euclidean distance. This analysis is much simpler than PLS as we only examine changes in correlation strength between two conditions. Here we selected the two conditions that showed the greatest differences from the PLS analyses and simply subtracted their correlation matrices (e.g., depression dataset: resting 1 vs. induced rumination). When doing so, we again found no relationship between Euclidean distance and changes in functional connectivity between the two conditions (Fig. 5).

It is possible that we observed no systematic relationship between saliences or correlation differences and Euclidean distances because neither saliences nor correlation differences measure the statistical reliability with which a functional connection changes. For instance, saliences are weighted coefficients that represent the magnitude of change. In the PLS framework, the statistical significance of a task effect is assessed at the level of the entire multivariate connectivity profile, but the significance of the task effect on individual functional connections is not tested directly.

**Figure 6:**
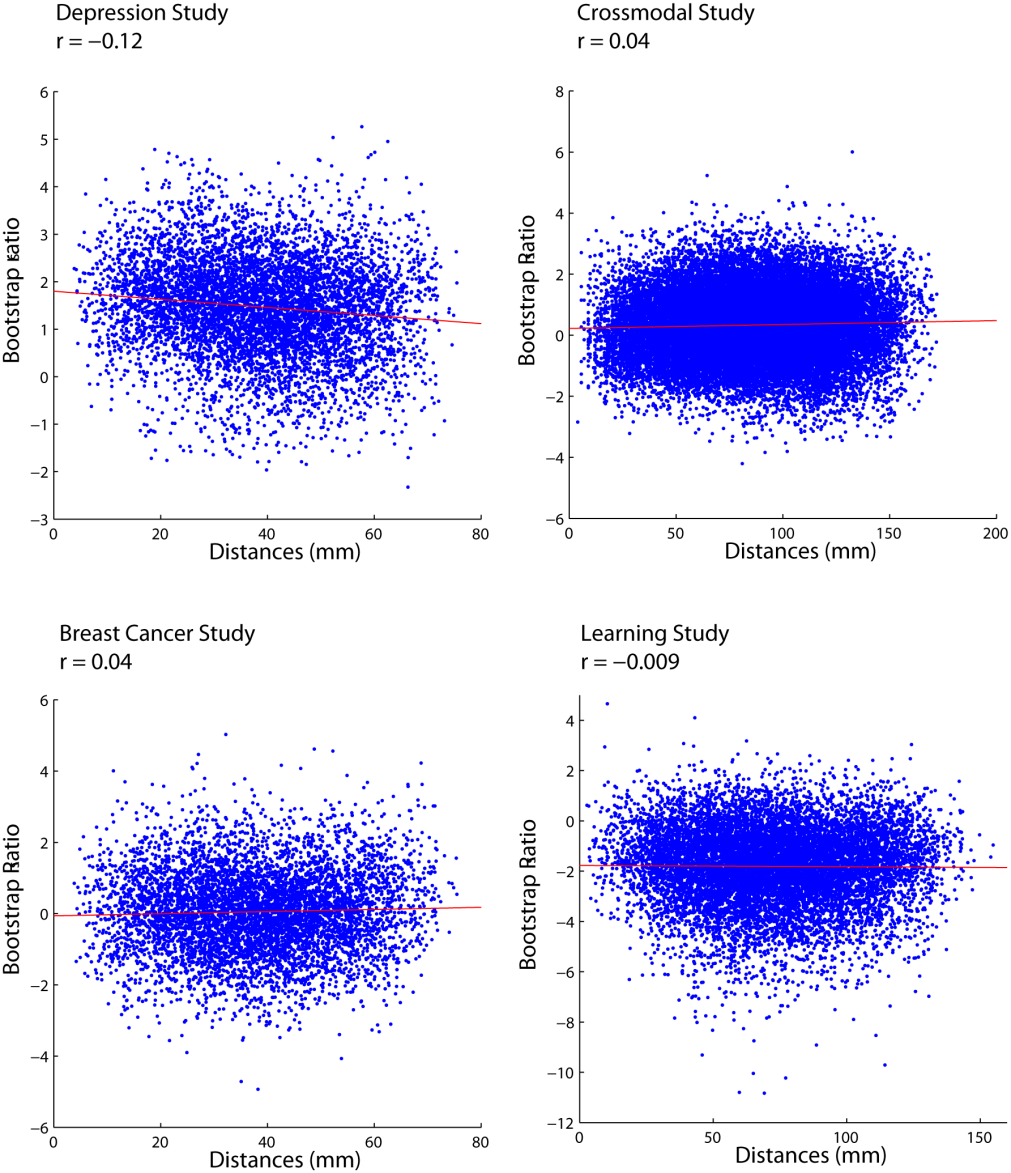
PLS bootstrap ratios for each functional connection are plotted against the Euclidean distance of that connection.

To address this problem, we related anatomical distance with the bootstrap ratio associated with each functional connection (Fig. 6). Bootstrap ratios are calculated by taking the ratio of the salience (which reflects the magnitude of a statistical effect) and the bootstrap-estimated standard error of that salience (which reflects the reliability of the salience). Thus, a high-valued bootstrap ratio for a given functional connection indicates both a strong contribution to the latent variable as well as reliability across subjects. Functional connections with large bootstrap ratios show a statistically significant propensity to change due to experimental manipulation. We then correlated the bootstrap ratios with Euclidean distances for each of the four studies, and again found no evidence for a systematic relationship (Fig. 6).

### Assessing statistical significance

The results in Figs. 4, 5 and 6 show no obvious relationship between Euclidean distance and changes in functional connectivity, but this is difficult to assess using conventional parametric statistical inference because the data points are dependent. For instance, functional connections are derived from correlation coefficients and are inherently non-independent. Thus, if one possesses knowledge of r(a, b) and r(a, c), it is possible to place limits on the value of r(b, c). As a result, the assumption of independence on which conventional inferential statistics are predicated would be violated, resulting in inflated p-values.

To address this problem, we estimated the p-value for the relationship between distance and change in functional connectivity with respect to a nonparametric null distribution, constructed using independent pairs of distances and correlations, e.g. r(a, b), r(c, d) and r(e, f) but not r(a, c), etc. For a parcellation with *n* nodes, there exist floor (*n*/2) such independent pairs. For a given set of independent pairs, we computed the correlation coefficient between distance and changes in functional connectivity. We then repeated the procedure 1000 times, to construct a sampling distribution of independent correlation coefficients. P-values were calculated as the proportion of independent pairs that were greater than or less than zero. Table 2 displays the mean, standard error and p-value associated with the correlations between salience and anatomical distance, for each of the four studies. The mean correlation values (rho) were low, with small standard errors (SEs). Importantly, one-tailed tests for rho<0 and rho>0 were non-significant, suggesting no evidence of a statistically significant relationship between change in functional connectivity and anatomical distance.

**Table 2:** For each study, a sampling distribution of correlations between changes in saliences and Euclidean distances was constructed using only independent pairs of nodes.

fMRI Study	rho	SE	P-value (rho>0)	P-value (rho<0)
**Depression**	–0.05	0.004	0.34	0.66
**Breast Cancer**	0.03	0.004	0.57	0.43
**Crossmodal**	0.05	0.003	0.71	0.29
**Learning**	–0.03	0.003	0.42	0.58

The table displays the mean correlation (rho) and standard error (SE) from this sampling distribution, as well as P-values associated with one-tailed tests for rho<0 and rho>0.

**Figure 7:**
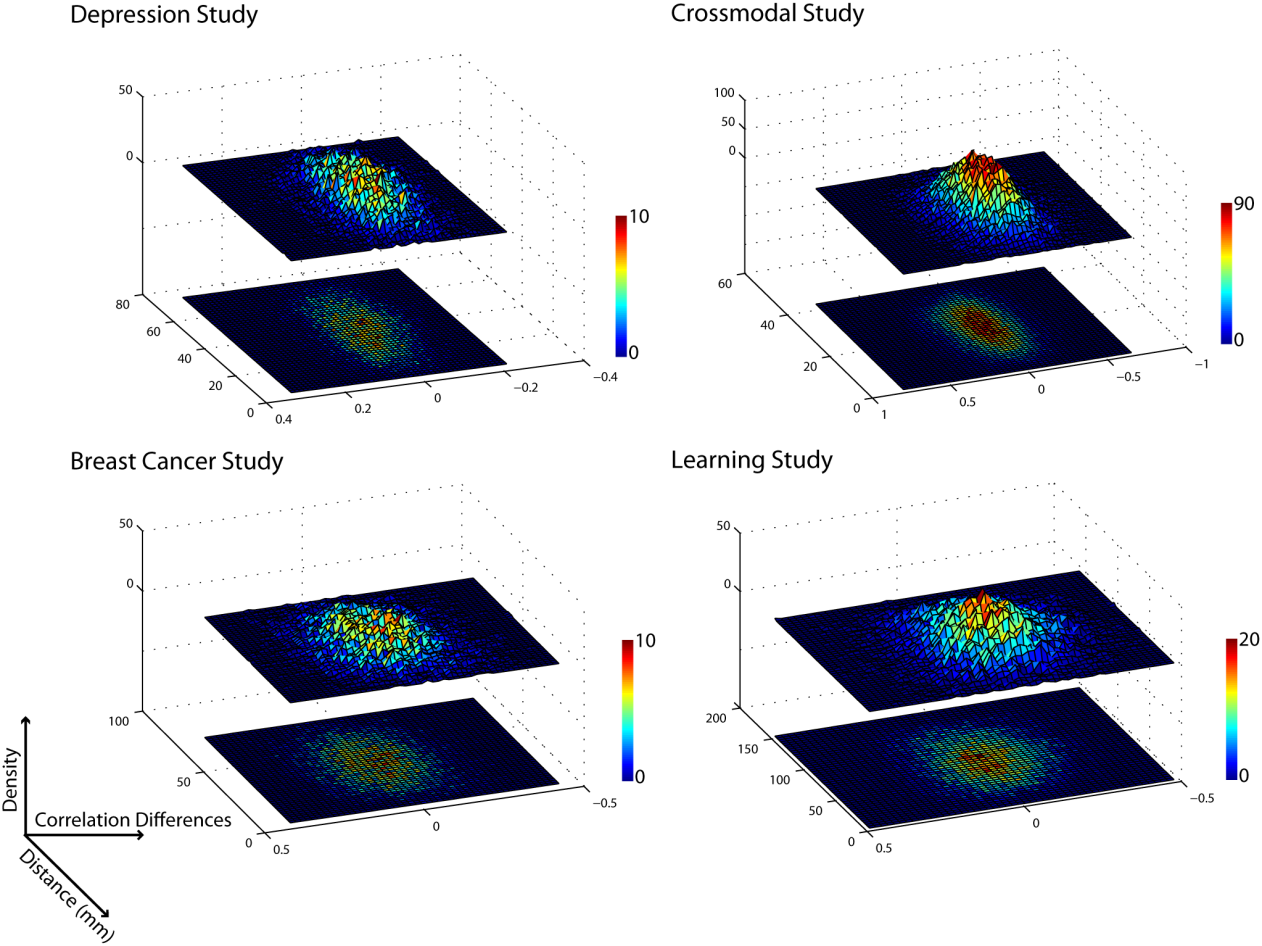
3-dimensional histogram of correlation differences by Euclidean distance for each study. In the 3-dimensional histogram (top) the number of observations (i.e., density) is represented by color and height. In the 2-dimensional plot (bottom) density is represented by color only.

To further illustrate that there is no evidence that these correlations are different from zero, we performed three more analyses. First, to ensure that there were no systematic trends in the dense portions of the plots shown in Fig. 4 and Fig. 5, we re-plotted the changes in correlation data with a 3-dimensional surface plot. As shown in Fig. 7, the areas of the plot with more data points do not show any relationship between changes in correlation and Euclidean Distance.

**Figure 8:**
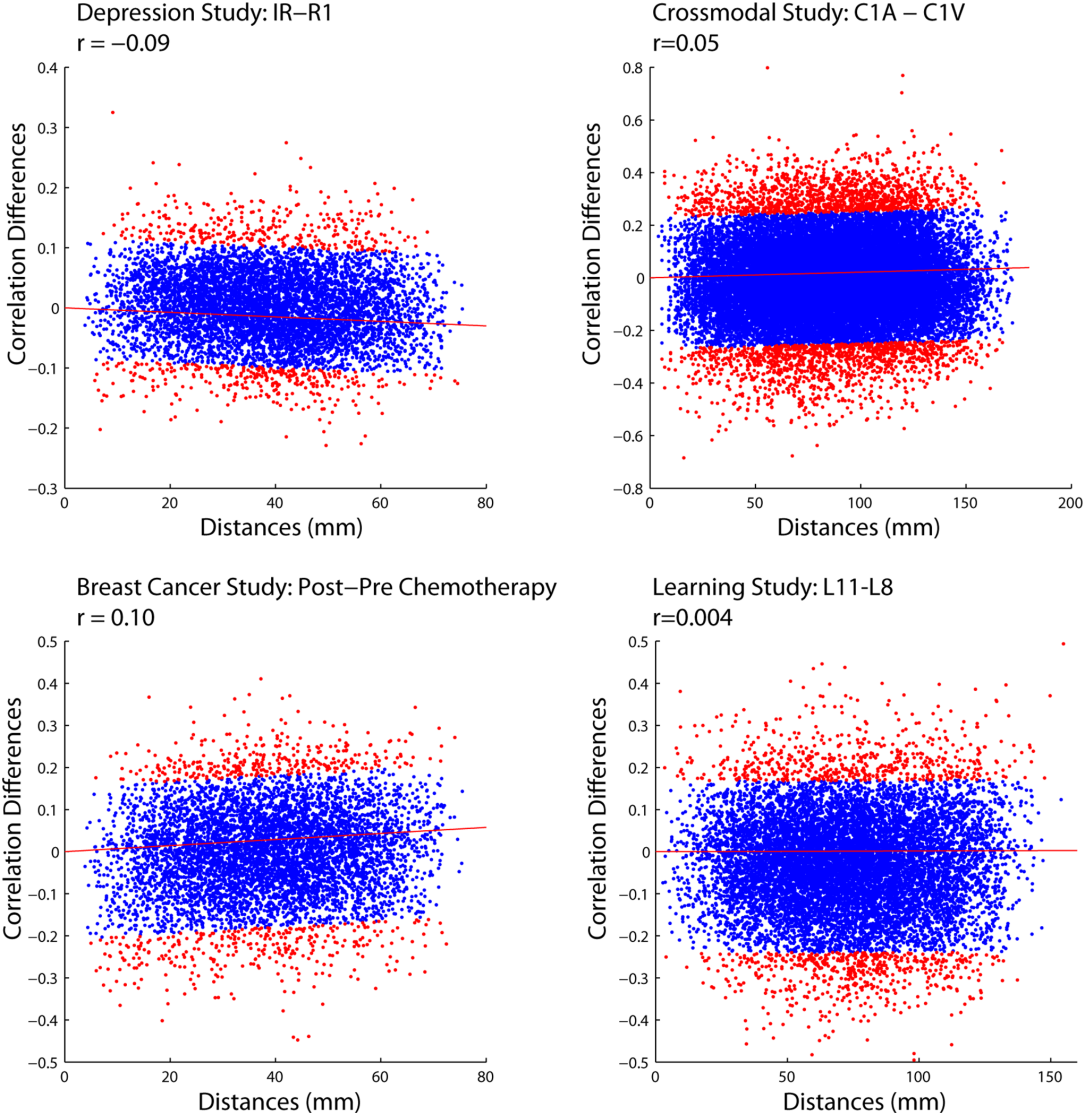
PCA analysis of scatter plots displaying the relationship between correlation differences and Euclidean distance. The PCA analysis was used to determine if outlying points exhibited a different relationship with Euclidean distance than the majority of the points. Points that were in the upper or lower 5% in terms of displacement from the primary axis of variance are colored red and suggest that there is no systematic relationship between correlation differences and Euclidean distances for the outlying points. The red line represents the primary axis of variance.

Second, a principal components analysis (PCA) was conducted as described by [28], to examine possible trends between Euclidean distance and correlation differences in the outlying points. To do so, we performed a PCA on the correlation differences versus Euclidean distances, and for each observation we calculated its displacement from the primary axis of variance. Points that were in the upper or lower 5% in terms of displacement from the primary axis of variance are colored red (Fig. 8). When examining these outlying points, there appears to be no systematic relationship between correlation differences and Euclidean distances.

**Figure 9:**
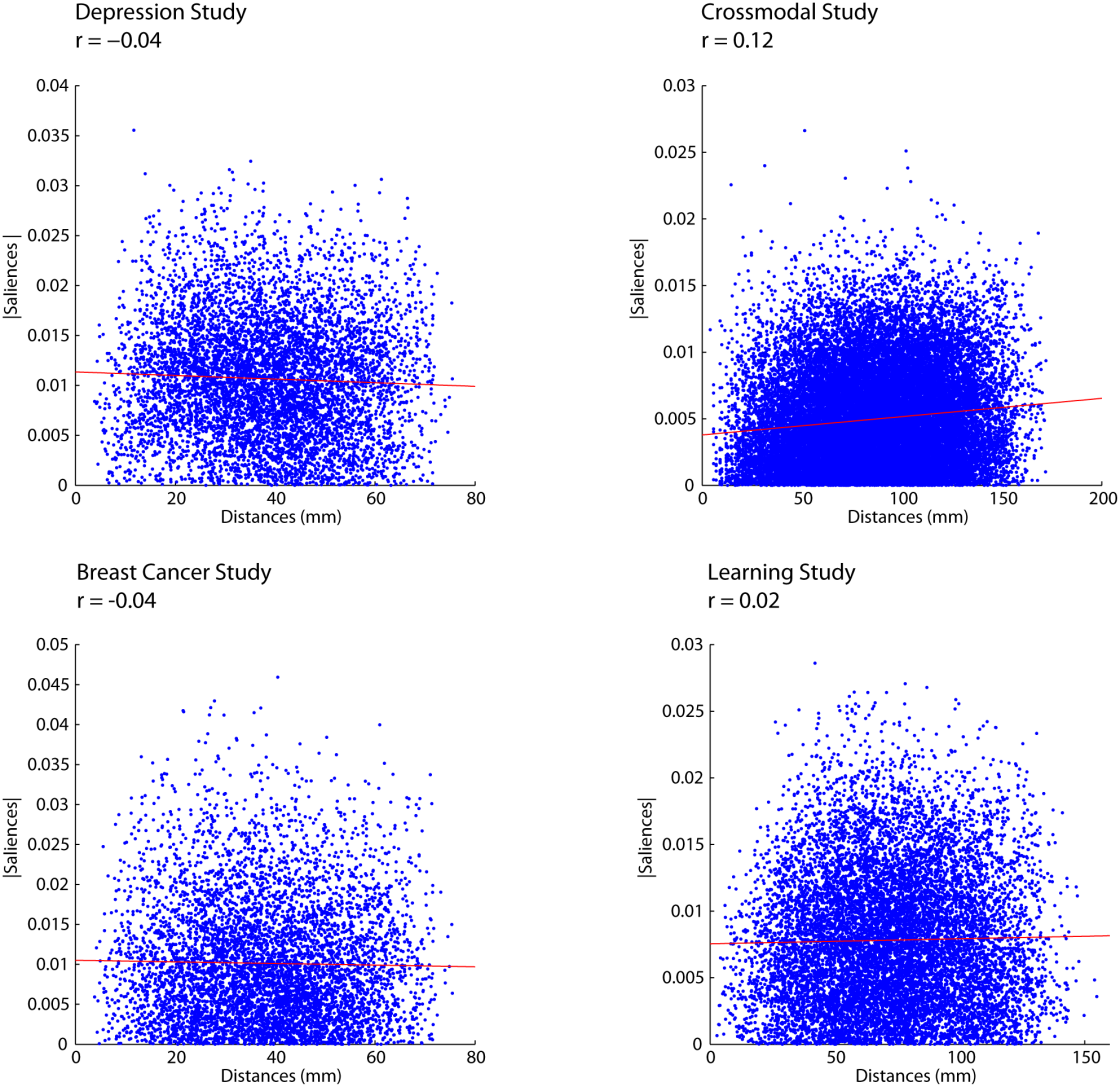
Absolute value of the Saliences for each of the four fMRI studies plotted against Euclidean distance.

**Figure 10:**
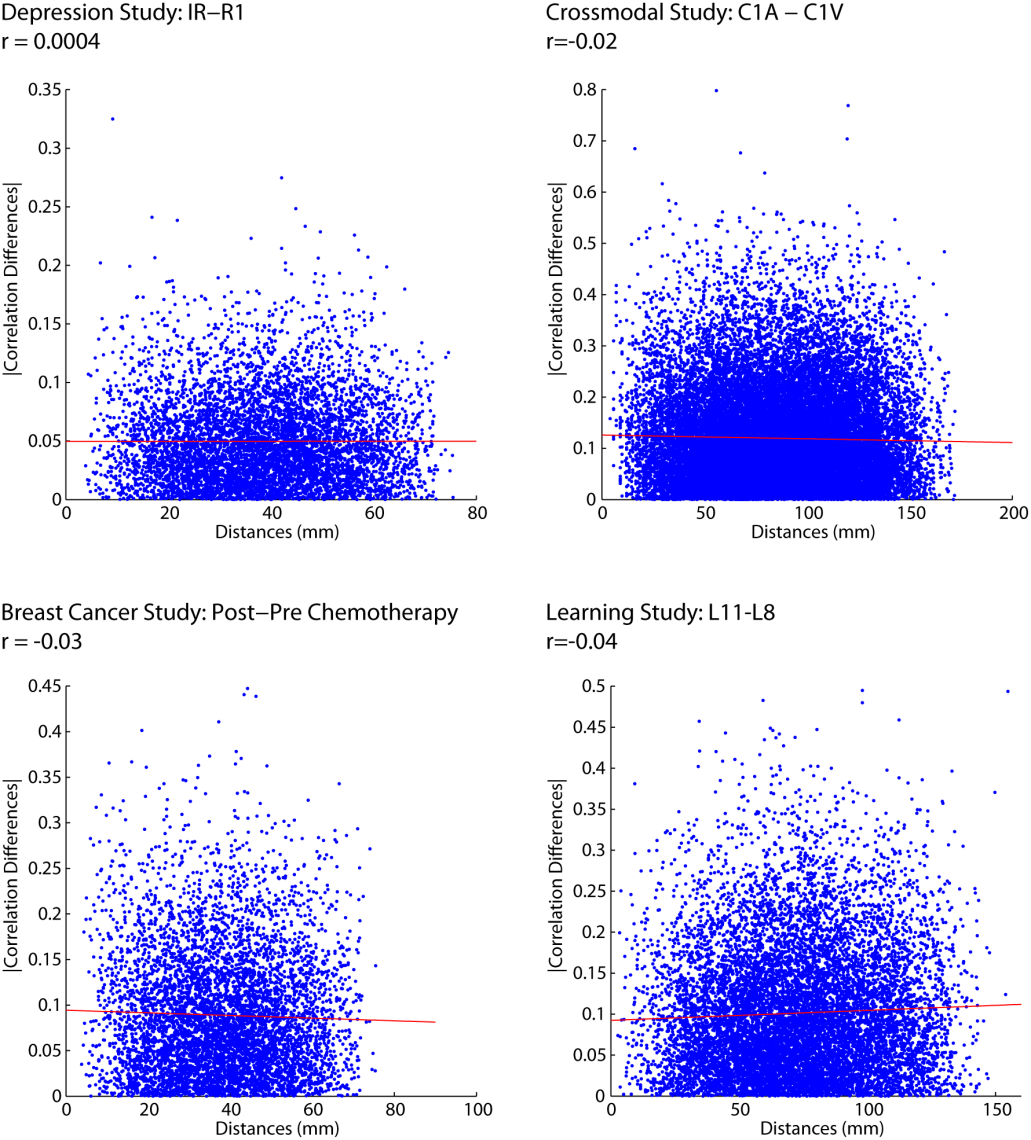
Absolute value of the correlation differences for each of the four fMRI studies plotted against Euclidean distance.

Third, we were concerned that using the signed value (i.e., positive or negative) of the salience or correlation change could impact the relationship between Euclidean distance and the magnitude of the effect. We calculated the absolute value of the salience and the absolute value of the change in correlation and found that the absolute value of the effects were unrelated to Euclidean distance (Figs. 9 and 10).

In summary, across different groups (healthy, depressed and breast cancer patients) and across different experimental conditions (learning task, crossmodal task, rest) we find the same results. Namely, we consistently find no evidence of a relationship between anatomical distance and changes in functional connectivity. This indicates that across different environmental conditions and groups, the functional connectivity of the whole brain is equally likely to change, independent of the distance between areas.

### Functional Specialization and Modularity

The previous set of analyses have shown that functional connectivity changes cannot be predicted by the distance of the functional connection across a wide range of populations, tasks, fMRI scanners, preprocessing parameters and parcellation schemes. However, it is possible that the relationship between proximity and functional connectivity change may be influenced by the modular organization of functional networks (Meunier, Lambiotte and Bullmore, 2010). For example, long-distance functional connections may be more likely to change between conditions if they connect regions belonging to different modules, as opposed to the same module.

**Figure 11:**
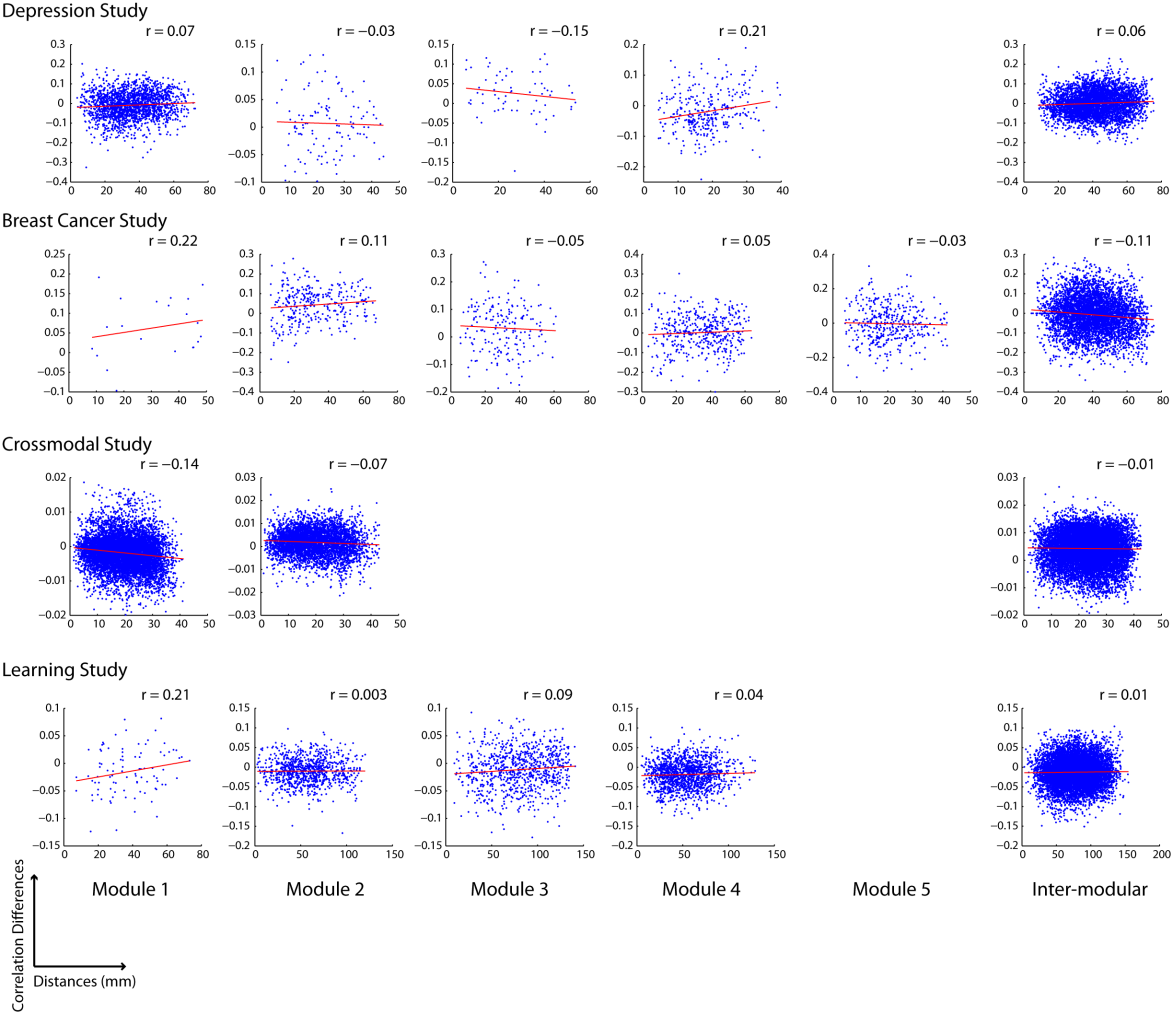
Relationship between Euclidean distance and Salience for functional connections within and between modules.

We applied a community detection algorithm to partition functional brain networks into modules [29], [30], allowing us to analyze how functional connections change both within and between modules. Specifically, we used the Louvain algorithm, as implemented in the Brain Connectivity Toolbox (Rubinov & Sporns, 2010). Following 100 runs on each data set, we selected the partition with the highest modularity, Q. We found no evidence of a relationship between functional connectivity changes and Euclidean distance either between or within modules (Fig. 11). In other words, the propensity of a functional connection between two areas to change does not appear to be influenced by the community membership of those areas

### Replication across multiple neuroimaging modalities

**Figure 12:**
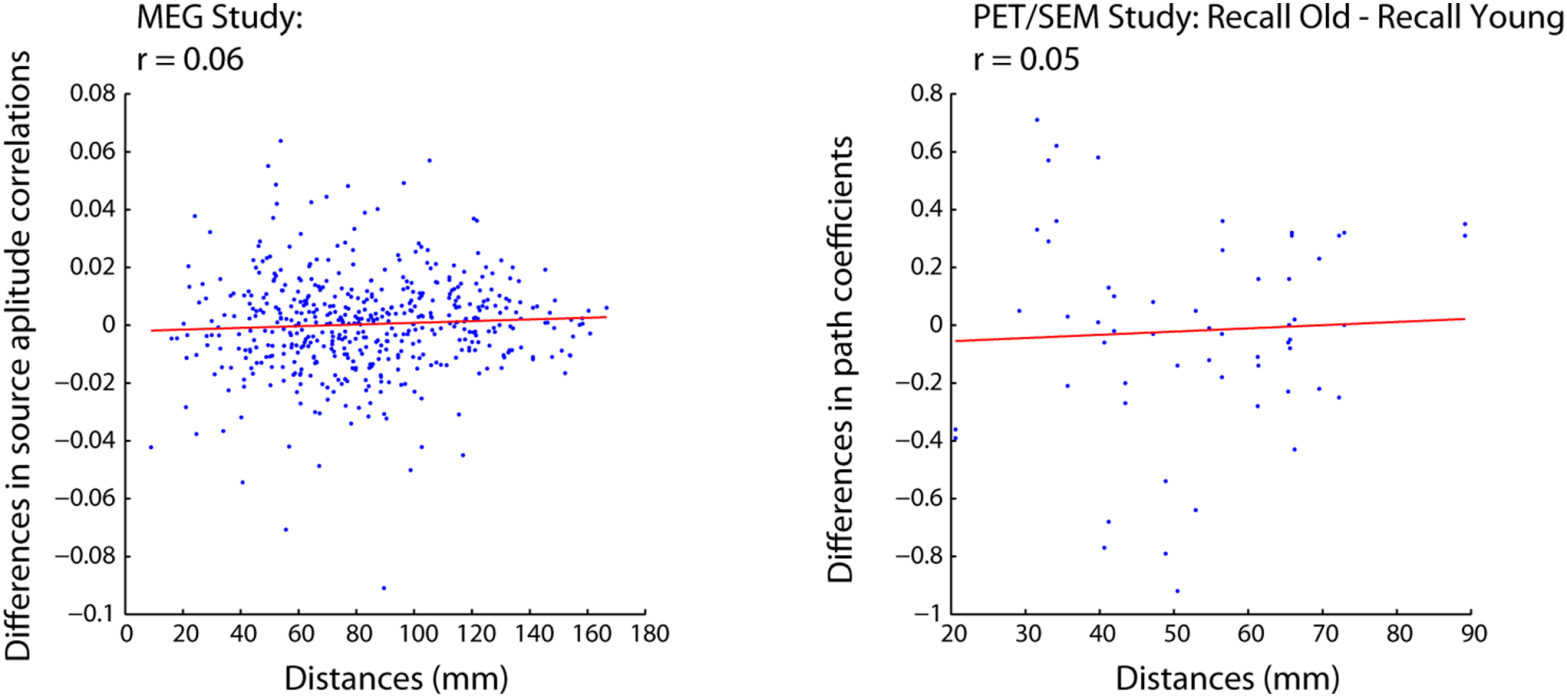
Relationship between changes in coherence and Euclidean distance for the MEG dataset (left) and differences in path coefficients from an SEM analysis of the PET dataset (right).

Our analyses thus far have focused on a single neuroimaging modality (i.e., fMRI). To examine whether these effects are idiosyncratic to fMRI, we analyzed a MEG dataset [18] and a PET dataset [31] that was published at an earlier time utilizing Structural Equation Modeling (SEM) [32]. In analyzing the MEG dataset, we replicated our previous results and found no relationship between changes in functional connectivity and Euclidean distance (Fig. 12).

We found the same pattern of results for the PET SEM dataset, where significant condition changes in path coefficients were not distance dependent (Fig. 12). It is noteworthy that the path coefficients in SEM represent effective connections, i.e. causal relationships based on known direct anatomical projections. In contrast to functional connections based on zero-order correlations, they are not confounded by potential indirect connections from other areas. This is important because our previous results with functional connectivity could be influenced by other indirect connections. The fact that similar results are observed for SEM suggests that the effects that we report are unlikely to be influenced by indirect connections.

### Intra- and inter-hemisphere connections

**Figure 13:**
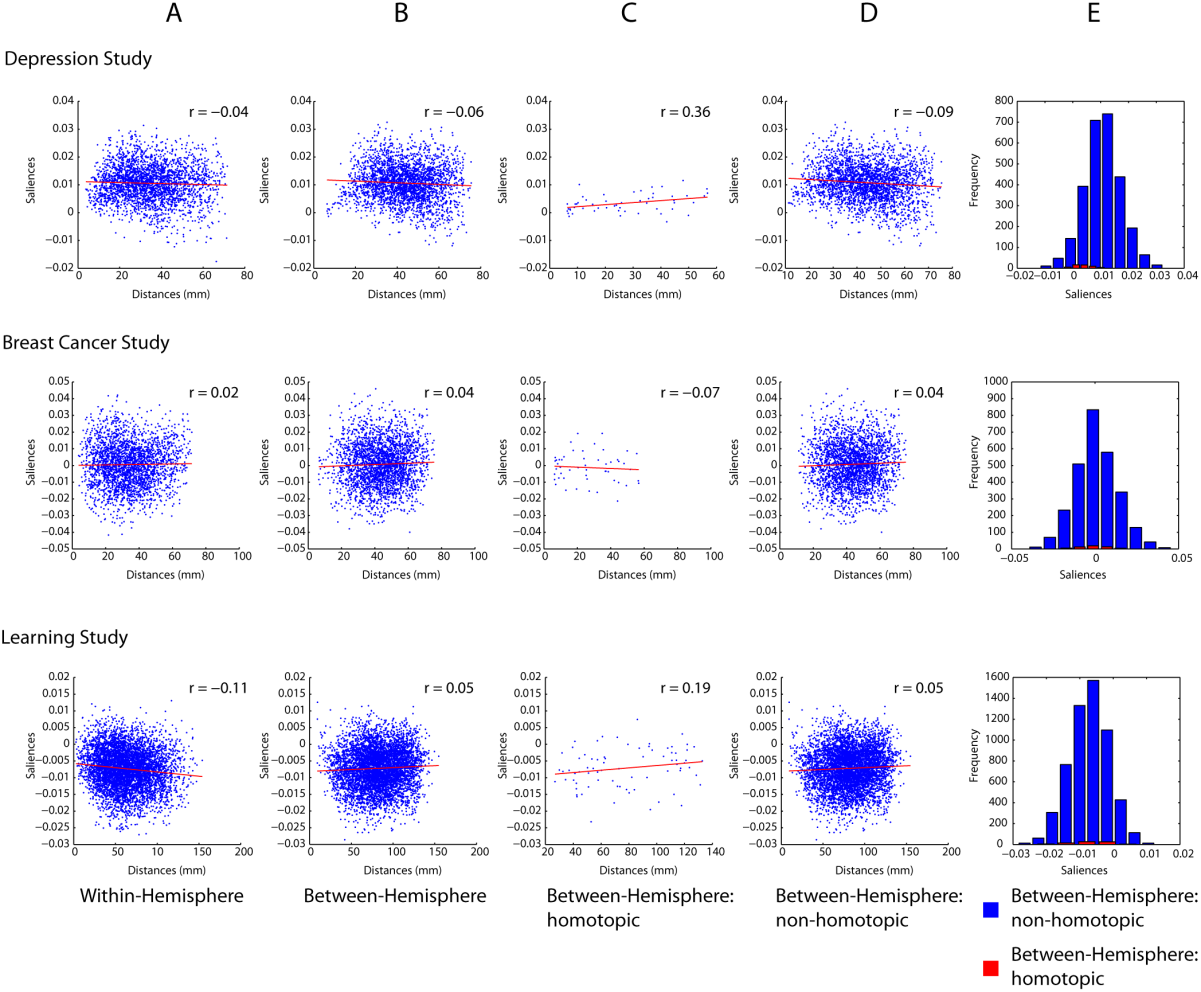
Relationship between changes in functional connectivity for nodes within a hemisphere (A), between hemispheres (B), between homotopic areas (C) and between non-topic areas (D). (E) Histogram showing the changes in functional connectivity for homotopic and non-homotopic areas.

Finally, we investigated whether spatial proximity had any differential effect on communication within and between hemispheres. For this analysis all functional connections were divided into two classes: intra-or inter-hemispheric. Inter-hemispheric connections were further stratified into those between homotopic and those between non-homotopic brain regions. Fig. 13 shows that even when functional connections are stratified in this way, distance did not predict changes in functional connectivity for either inter- or intra-hemispheric functional connections (Fig. 13A, B).

Interestingly, the relationship between Euclidean distance and change in inter-hemispheric functional connectivity did appear to be affected by whether connections were between homotopic areas. When inter-hemispheric functional connections were further segregated into homotopic and non-homotopic connections, there was still no reliable relationship between changes in functional connectivity and distance (Fig. 13C, D). However, functional connections between homotopic brain areas were associated with smaller change saliences that deviated from zero far less than non-homotopic connections, indicating that they were less likely to change and were more stable than functional connections between non-homotopic areas (Fig. 13D). This is illustrated further in Fig. 13E, which shows the histogram of change saliences for homotopic and non-homotopic functional connections. The homotopic functional connections deviate from zero considerably less than non-homotopic connections.

To statistically assess whether homotopic functional connections were more stable compared to non-homotopic connections, we combined the change saliences for both types of connections and calculated the proportion of each connection type that exceeded the 95% confidence interval of change saliences. We found that, for each study, the proportion of change saliences that exceeded the 95% confidence interval was greater for the non-homotopic connections compared to the homotopic connections. For the Depression, Breast Cancer and Learning studies, these proportions, for the non-homotopic and homotopic connections respectively, were: 5.4% vs. 1.9%, 5.8% vs. 0% and 5% vs. 3.9%. This provides an interesting addition to previous reports, which have shown stronger functional connectivity between homotopic areas compared to functional connectivity between non-homotopic areas [19], [33], [34].

## Discussion

In the present report we investigated whether functional connectivity stability could be predicted by anatomical distance. The goal was to determine whether one of two hypotheses were true: 1) That long-range connections were more likely to change with different manipulations or 2) that all connections, independent of distance, were equally likely to change with different manipulations (i.e., equivalent stability). We implemented a multivariate approach to test these hypotheses, allowing us to simultaneously examine changes in all functional connections in the brain.

We found three important results. First, within a single condition, Euclidean distance significantly predicts functional connectivity; namely, regions that are closer together have stronger functional connectivity and regions further apart have weaker functional connectivity (Fig. 2). We found this result to be robust across different tasks, populations, preprocessing parameters and parcellation schemes. This result is also consistent with previous reports in the literature [2], [15], [19].

Second, we find that anatomical distance is not a significant predictor of the stability of functional connectivity (Figs. 4–9 & 11), meaning that changes in functional connectivity are not specific to short- or long-range connections; thereby lending support for hypothesis 2. These effects were also robust for a wide range of tasks, populations, preprocessing parameters, parcellation schemes and neuroimaging modalities, suggesting that these effects are not idiosyncratic to particular experimental and analysis parameters.

Third, we find that homotopic functional connections appear to be more stable than any other type of connection. This finding complements previous reports that homotopic functional connections are stronger than other types of functional connections [19], [33], [34]. We find that functional connections between homotopic brain areas are most stable, and therefore least likely to change.

It is important to consider whether the main result reported here – the weak or non-existent relationship between distance and functional stability – is a “null” result and whether such a finding holds scientific value. Standard inferential statistics do not allow us to rule out such a relationship; rather, they can only indicate that there is no significant evidence to suggest the existence of such a relationship. The present analyses demonstrate that this “null” result is remarkably consistent and robust. First, the independent pairs analysis reported in Table 2 shows that the miniscule correlations between functional connectivity change and anatomically distance are statistically stable, with tight confidence intervals. Second, the finding is robust and persistent across six different data sets that were deliberately chosen to cover a wide range of populations and brain states. Altogether, the present investigation supports one particular hypothesis: that all functional connections, regardless of their distance, are equally likely to change.

The fact that all functional connections are equally likely to change, regardless of their length, is counterintuitive. Namely, one might expect connections between proximal areas to be more stable given that proximal brain areas tend to be functionally more similar and highly correlated within-condition (Fig. 2). While somewhat counterintuitive, the fact that long- and short-range connections are equally stable may support the balance between local segregation (i.e., the isolation of specialized processing) and global integration (i.e., the combination of different processes) in the brain [12], [35]. A functional connectivity landscape in which short-range and long-range connections are equally likely to be strengthened or weakened may facilitate fluid transitions between regional specialization and global integration, thereby allowing the brain to explore a wider range of functional network configurations. This would appear to be a desirable and an adaptive quality, as it would facilitate a more diverse repertoire of cognitive processes.

As mentioned above, one variable that was predictive of functional connectivity change was whether a connection was between homotopic areas. Connections between homotopic areas did not change significantly for changes in task. The source of this stability is likely to be the inter-hemispheric callossal fibers connecting these homotopic regions [19]. This small subset of connections remained relatively stable against a perpetually changing functional connectivity landscape. This stability, combined with increased functional connectivity for those connections [19], [33], [34], may serve as a grounding principle to maintain steadiness in brain networks in the face of changes in the environment.

Our results show a strong negative relationship between functional connectivity and anatomical distance, but no relationship for changes in functional connectivity and anatomical distance. One may wonder if changes in functional connectivity do not correlate with anatomical distance because of some type of statistical artifact. Namely, within-condition functional connectivity has a robust negative relationship with anatomical distance, and it may be the case that the difference between two such negative trends automatically results in a “flat”, non-significant trend as observed in these data. This explanation is highly unlikely because it would imply that the changes in individual data points between two conditions are non-systematic and completely random, whereas our PLS results show that the changes in functional connectivity between conditions are statistically significant and highly reliable across participants. As such, we consider it unlikely that these results are driven by this type of statistical artifact.

One possible limitation of the present study is that we use Euclidean distance as a proxy for anatomical distance. The former may not be completely representative of the lengths spanned by anatomical projections due to the well-known folding of cortical tissue. Note, however, that we find the relationship between changes in functional connectivity and Euclidean distance to be the same for long and short distances. Additionally, if we look only at relatively long distances that would be unaffected by folding, we still find no relationship between Euclidean distance and changes in functional connectivity.

In conclusion, the present investigation presents the first detailed report that the changes in cognitive state are supported by long-range and short-range functional connections in equal measure. At the same time, connections between homotopic areas are among the most stable, which may provide stability in the face of a changing environment. These may be fundamental brain phenomena to strike a balance between local segregation and global integration.

## Materials and Methods

### Partial least-squares (PLS) Analysis

These analysis parameters are the same as those presented in [16]. Whole-brain fMRI timeseries were parcellated into 116 brain areas based on the AAL template [36] for the depression and breast cancer datasets, and with the template (that aggregated across four atlases) for the Learning dataset. For the Crossmodal dataset every 100^th^ voxel in gray matter was used in the analysis. These timeseries were then correlated together to form a full correlation matrix for all regions correlated with all other regions for each participant and condition.

Partial least-squares (PLS) analysis is a multivariate statistical analysis that is used to relate two sets of variables together. In the case of neuroimaging one set of variables could be brain data (e.g., BOLD signal per voxel per time point) while the other set of variables could be the experimental study design (e.g., groups and experimental conditions). The first step in PLS is to compute the covariance between the two sets of variables (i.e., the “cross-block” covariance). The second step of PLS is to perform a singular value decomposition on the “cross-block” covariance matrix to determine the combination of variables in each set that are optimally related to each other (i.e., that accounts for the greatest proportion of “cross-block” covariance). This combination, termed a latent variable (LV), is comprised of a linear combination (i.e., weighted) of variables from both sets (i.e., brain and experimental sets), as well as a scalar singular value. For the brain set this combination is a spatial-temporal pattern (saliences) and for the design set this combination is a contrast between groups and conditions. The mutually orthogonal LVs are extracted in order of magnitude, i.e. the first LV explains the most “cross-block” covariance, the second LV explains the second most “cross-block” covariance, etc. In the present study the brain data were not activations (i.e., voxels in time), but rather were functional connections between the parcels. This produced 6670 unique connections for the AAL parceled data (i.e., (116*115)/2). As a result, the latent variable represents a weighted combination of functional connections that optimally relate to our groups and conditions (e.g., for the depression dataset: depressed vs. control for the 4 different experimental conditions).

The significance of each LV is assessed with permutation testing. A set of 500 permuted samples were created by randomly re-ordering participants and condition labels without replacement for the brain set (note: groups and conditions are permuted because PLS is uncovering the interactions between group and condition effects) while the labels for the design set are maintained resulting in 500 new covariance matrices. These covariance matrices embody the null hypothesis and are then each subjected to singular value decomposition as before resulting in a null distribution of singular values. The significance of the original LV is assessed with respect to this null distribution; the p-value is calculated as proportion of permuted singular values that exceed the original singular value.

The reliability with which each functional connection expresses the LV pattern is determined with bootstrapping. A set of 500 bootstrap samples are created by re-sampling participants with replacement within each condition (i.e., preserving condition labels, but not participant labels). Each new covariance matrix is subjected to singular value decomposition as before and the saliences of the bootstrapped dataset are used to build a sampling distribution of the saliences from the original dataset. The purpose of a constructed a bootstrapped sampling distribution is to determine the reliability of each salience; saliences that are highly dependent on which participants are included in the analysis will have wide distributions. A single index of reliability (“bootstrap” ratio) is calculated by taking the ratio of the salience to its bootstrap estimated standard error. A bootstrap ratio for a given functional connection is large when that functional connection has a large and stable salience.

### Depression Dataset Details

The methods for this depression dataset are the same as those from [16].

#### Participants

Sixteen participants diagnosed with clinical depression (mean age = 26.6 std. age = 6.12, 11 female) and seventeen non-depressed controls (mean age = 24.4 std. age = 5.83, 13 female) participated in our study. Participants’ diagnosis of MDD vs. a non-diagnosis was determined by a trained clinician administering the Structured Clinical Interview Diagnostic (SCID) IV [37]. All participants provided written informed consent in accordance with the Institutional Review Board of the University of Michigan, which approved this study. Participants were compensated $25/hour for their participation.

#### fMRI Acquisition and Analysis parameters

Images were acquired on a GE Signa 3-Tesla scanner equipped with a standard quadrature head coil. Functional T2* weighted images were acquired using a spiral sequence with 40 contiguous slices with 3.44×3.44×3 mm voxels (repetition time (TR) = 2000 ms; echo time (TE) = 30 ms; flip angle = 90°; field of view (FOV) = 22 cm). A T1-weighted gradient echo anatomical overlay was acquired using the same FOV and slices (TR = 250 ms, TE = 5.7 ms, flip angle = 90°). Additionally, a 124-slice high-resolution T1-weighted anatomical image was collected using spoiled-gradient-recalled acquisition (SPGR) in steady-state imaging (TR = 9 ms, TE = 1.8 ms, flip angle = 15°, FOV = 25–26 cm, slice thickness = 1.2 mm).

Each SPGR anatomical image was corrected for signal in-homogeneity and skull-stripped using FSL’s Brain Extraction Tool [38]. These images were then segmented with SPM5 (Wellcome Department of Cognitive Neurology, London) into grey matter, white matter and cerebrospinal fluid and normalization parameters for warping into MNI space were recorded.

Functional images were corrected for differences in slice timing using 4-point sinc-interpolation [39] and were corrected for head movement using MCFLIRT [40]. To reduce noise from spike artifacts, the data were winsorized prior to normalization [41] by exploring time courses for each voxel and finding values that were 3 standard deviations (SDs) away from the mean of that voxel’s time course. Spikes that were above 3 SDs from the mean were made equal to the mean +3 SDs and spikes that were 3 SDs below the mean were made equal to the mean –3 SDs. The segmented normalization parameters were then applied to the functional images maintaining their original 3.44×3.44×3 mm resolution and they were spatially smoothed with a Gaussian kernel of 8 mm.

To correct for physiological artifacts, all of our functional data were corrected using the PHYCAA algorithm. This is an adaptive multivariate model that estimates and removes physiological noise components from fMRI data, without requiring external measures of heartbeat and respiration [42].

Furthermore, 24 motion parameters were calculated, which included the linear, squared, derivative, and squared derivative of the six rigid-body movement parameters [43]. A principal component analysis was performed on these 24 motion parameters and only the first principal component, which accounted for nearly 90% of the motion variance, was covaried out from each voxel’s time course to remove any signal that could be attributed to motion (Berman et al., 2013). Lastly, functional images were parceled into 116 different ROIs based on the AAL template [36] for analysis.

#### Task Parameters

Participants initially performed two resting-state scans back-to-back that were 8 minutes in length. Participants were instructed to look at a fixation cross at the center of the screen and were told not to think about any particular thought (i.e., they could think about whatever they wanted to). After acquiring anatomical images of the brain, participants were then taken out of the scanner and were asked to generate four negative autobiographical memories. In order to facilitate the generation of the memories, we provided four distinct prompts such as “Please recall a specific time when you were rejected by someone you loved or still love” or “Please recall a specific time when you were very embarrassed.” Following such a prompt, participants had to indicate whether they were able to think of a prompted event and if they were successful in doing so they were asked to re-live the event for 30 s as well as they could. Next, they had to rate how positively and negatively they felt about that event using a visual-analog scale. Finally, they were asked to describe the event in a few sentences and to create a 2 to 3 word long cue that reminded them of the event.

Following the creation of these events, participants went back into scanner. Now only considering the two most negatively rated events, we simultaneously showed the event description and its cue and instructed participants to pair the two so that they were able to easily recall the memory after seeing the corresponding cue. In a next step, participants were asked to practice recalling the events after seeing the corresponding cue. For this purpose, participants had to press a key as soon as they were able to bring to mind the cued event. The cues were repeated until participants were able to vividly recall the corresponding event in less than 5 s.

Following this practice session, the induction period started. Participants were presented with the cue of one of the two memories that they rated as being most negative. The cue stayed on the screen for 3 min and participants were instructed to re-live that experience as vividly as possible in their imagination and to go back to the time and place of the experience in their imagination and to re-experience it happening to them all over again. After 3 min the cue of the second negatively rated event was presented on the screen. After this negative mood induction, participants again performed another resting-state scan where they fixated on centrally on a fixation cross for 8 minutes.

### Breast Cancer Dataset Details

Similar methods with the same participants performing a verbal working memory-task can be found in [26], [27].

#### Participants

Sixteen women (mean age = 49.3, std. = 10.7) with breast cancer that underwent chemotherapy, sixteen women (mean age = 55.0, std. = 8.9) with breast cancer who underwent radiation therapy and sixteen aged-matched non-cancer controls (mean age = 51.4, std. = 7.9) participated in our study. Participants were tested before and after treatment. For the non-cancer controls an equal amount of time separated their two imaging sessions, which was approximately 5 months. All participants provided written informed consent in accordance with the Institutional Review Board of the University of Michigan, which approved this study.

#### fMRI acquisition parameters

Images were acquired on a GE Signa 3 Tesla scanner equipped with a standard quadrature head coil. Functional T2* weighted images were acquired using a spiral sequence with 25 contiguous slices with 3.75×3.75×5 mm voxels (repetition time (TR) = 1500 ms; echo time (TE) = 30 ms; flip angle = 70°; field of view (FOV) = 24 cm). A T1-weighted gradient echo anatomical overlay was acquired using the same FOV and slices (TR = 225 ms, TE = 5.7 ms, flip angle = 90°). Additionally, a 124-slice high-resolution T1-weighted anatomical image was collected using spoiled-gradient-recalled acquisition (SPGR) in steady-state imaging (TR = 9 ms, TE = 1.8 ms, flip angle = 15°, FOV = 25–26 cm, slice thickness = 1.2 mm).

#### fMRI Preprocessing Parameters

Each SPGR was corrected for signal in-homogeneity and skull-stripped using FSL’s Brain Extraction Tool [38]. These images were then normalized with SPM5 (Wellcome Department of Cognitive Neurology, London); the normalization parameters for warping to the standard MNI template were recorded and applied to the functional images.

Functional images were corrected for differences in slice timing using 4-point sinc-interpolation [39] corrected for head movement using MCFLIRT [40]. To reduce noise from spike artifacts, the data were winsorized prior to normalization [41] by exploring time courses for each voxel and finding values that were 3 standard deviations (SDs) away from the mean of that voxel’s time course. Spikes that were above 3 SDs from the mean were made equal to the mean +3 SDs and spikes that were 3 SDs below the mean were made equal to the mean –3 SDs. After normalization the functional images were spatially smoothed with a Gaussian kernel of 8 mm.

Nuisance covariates were then regressed out of the data. These included the principal component of the estimated motion parameters (Berman et al., 2013), and the average global signal time course within brain voxels. Data were then low-pass filtered (cutoff of 0.08 Hz), in order to remove high-frequency noise and retain only the BOLD frequency band of interest. Finally, average ROI time courses were extracted from the data using the AAL template, generating 116 anatomically labeled ROIs of interest. These time courses were then used for cross-correlation analysis.

#### Task Parameters

Participants were instructed to attend to a centrally located fixation cross while in the fMRI scanner and instructed not to think about any particular memory or thought (i.e., they could think about whatever they wanted to).

### Learning Dataset Details

#### Participants

Sixteen healthy, right-handed, native English-speaking individuals were enrolled in the study. Participants were screened for neurological, psychiatric and relevant medical conditions by a study-Medical Doctor and were also assessed by the MINI-Plus [44].

All participants provided written informed consent in accordance with the Research Ethics Boards of Baycrest Hospital, the Centre for Addiction and Mental Health and the University of Toronto, which approved this study.

The final sample was comprised of data from twelve participants (5 females, mean age: 30.8±8.2). Data from four participants were excluded due to improper task performance, technical/equipment issues and/or excessive movement artifact during fMRI scanning.

#### fMRI Acquisition

MRI images were acquired on a Siemens Trio 3T magnet. A T1-weighted anatomical scan was obtained using SPGR (TE = 2.6 ms, TR = 2000 ms, FOV = 256 mm, slice thickness = 1 mm). The SPGR was co-registered with the functional images and was also used to rule out any gross brain anomalies. T2* functional images (TE = 30 ms, TR = 2000 ms, flip angle = 70°, FOV = 200 mm) were obtained using an echo-planar image (EPI) acquisition sequence leading to a blood oxygenation level-dependent (BOLD) contrast. Each functional sequence consisted of twenty-eight 5-mm-thick slices in the axial-oblique plane positioned to image the entire brain.

Participants’ responses were made with their right hands using the Fibre-Optic Response Pad (FORP) system, which has two four-button response pads and is designed for MR compatibility. Visual stimuli were presented on a rear projection screen placed at the foot of the MR scanner using an LCD projector. The participants viewed the stimuli using a mirror mounted on the head coil. Auditory stimuli were presented using the Silent Scan auditory presentation system (AVOTEC), which uses air conduction to transmit tones and headphones to attenuate the gradient noise. E-Prime Stimulus Presentation Software (http://www.pstnet.com) was used to control stimulus presentation, collect behavioral responses and to log the precise timing of the stimulus events.

#### fMRI Preprocessing Parameters

Functional images were slice-timed corrected with AFNI (http://afni.nimh.nih.gov/afni). Motion correction was completed using AIR (http://bishopw.loni.ucla.edu/AIR5/) by registering functional volumes to the 100th volume within each run. All functional volumes within each motion-corrected run were averaged to create mean functional volumes for each run. Using a rigid body transformation model, this mean functional volume was then registered with each participant’s structural volume. The structural data were spatially normalized to the Common Anatomical Template previously described by [45]. Thus, the end result was a direct nonlinear transform from each initial fMRI volume into the Common Template space.

Data were smoothed using a 7 mm Gaussian kernel. The voxel time series were further adjusted by regressing out motion correction parameters, white matter (WM) time series and CSF time series. As [46], the time series of the unsmoothed data from small regions-of-interest in the corpus callosum (WM) and ventricles (CSF) of the Common Template were used as the white matter and CSF regressors respectively. This last step minimizes contamination of WM and CSF time series that can occur when signals from neighboring gray matter voxels are mixed in due to spatial smoothing or registration errors. Furthermore, we performed high-pass temporal filtering using FSL (Gaussian-weighted least-squares straight line fitting, with sigma = 75 s).

We combined four published probabilistic atlases to create a gray matter atlas with 152 Regions of Interest (ROIs). Three of these atlases are part of the FSL distribution: Harvard Oxford-Cortical, Harvard Oxford-Subcortical and Oxford Thalamic Connectivity atlases. The fourth atlas was the SUIT Cerebellar atlas (www.icn.ucl.ac.uk/motorcontrol/imaging/suit.htm). Each of the four atlases provides a thresholded maximum probability ROI mask with a threshold of 25%. This means that each voxel within the 3D image space was assigned to exactly one ROI, namely the one with the highest probability according to the sample of manually delineated single participant ROIs. If this maximum probability was below 25%, then the voxel was not assigned to any ROI. In this way each atlas provided a discrete segmentation image where voxel intensity signifies the voxel’s most probable ROI membership.

All ROIs from four atlases were used, with the exception of global regions from the subcortical atlas, namely, Cerebral White Matter, Lateral Ventricles and Cerebral Cortex. To obtain consistent parcellation of thalamus we intersected a single thalamic ROI from the subcortical atlas with all thalamic subregions from the Thalamic Connectivity atlas. For a small number of voxels, there was a conflict in terms of ROI membership, such that a voxel could be a part of one ROI according to one atlas and also a part of another ROI according to another atlas (e.g. along cerebrum/cerebellum border). We resolved such conflicts by setting the following atlas precedence order from highest to lowest: Cortical, Subcortical, Thalamic, Cerebellar.

In order to create consistent left and right hemispheric subdivisions we combined ROIs from four atlases, some of which were lateralized and others which were not. For example, none of the regions within Cortical, Subcortical and Thalamic atlases are lateralized. On the other hand, the Cerebellum atlas has both lateralized and medial (non-lateralized) regions. To create a lateralization within the Cerebellum and brain stem, we used the midsaggital plane of the MNI152 template brain (x = 0 in world coordinates) and divided regions into left and right depending on the position with respect to the plane. We made an exception for medial cerebellar regions, which were left as such. The final combined gray matter ROI atlas consists of 152 regions: 48+48 cortical lateralized regions, 7+7 lateralized thalamic regions, 6+6 additional lateralized subcortical regions (pallidum, putamen, caudate, hippocampus, amygdala and accumbens), 10+10 lateralized cerebellar regions, 8 medial cerebellar regions and 1+1 lateralized brain stem regions.

For each of the 152 ROIs we extracted the time series as a weighted sum over the voxels within the region. The weighting was provided by the distance transform from the region’s border, so that voxels at the core of the region would be weighted more than the voxels near region’s border. The weight of each voxel was calculated as a squared distance from the region’s border, normalized by the sum of all squared distances. This weighting scheme was designed to reduce artificially high correlations between small neighboring regions where signal can be smeared due to coarse resolution, and preprocessing and registration errors. This was performed separately for each condition.

#### Task Parameters

The overarching goal for the experiment was for participants to learn a new lexicon comprised of 30 pseudowords in an associative learning task spanning two fMRI scanning sessions. The study occurred over a three-day period. On the first day participants were trained in a MRI simulator using a parallel set of stimuli to ensure full understanding of the task to be completed during fMRI scanning. FMRI scanning then occurred on two days over the course of a one-week period.

The learning task used was developed by [47], but modified for English speakers. Participants heard a spoken pseudoword in their headphones and then briefly saw a picture of an everyday object followed by a fixation cross. Sometimes the pairings were ‘correct’ and sometimes ‘incorrect’. Participants indicated whether or not they thought the pairings were correct by pushing one of two buttons on a response pad. The underlying learning principle was a higher co-occurrence of ‘correct’ pairings with a 20∶1 (correct:incorrect) by the end of both scanning sessions. The learning runs across both days were additive in that the vocabulary to be learned for each participant was comprised of the same 30 words-object pairings on each of the days.

Participants also completed a reaction-time task, which was identical in structure, but used another parallel set of stimuli and lacked an underlying learning principle. In this task, participants heard a pseudoword, and were then presented with an object-picture. They were asked to simply push the first button as quickly as possible when the picture appeared on the screen.

The two scanning sessions were identically structured. The functional runs began with the reaction-time task (120 trials), followed by five learning runs (900 trials in total) and then another reaction-time run (120 trials). Each run lasted approximately 6.5 minutes. Data used in this analysis are from the second scanning day and include the first reaction-time task and the subsequent five learning runs.

### Crossmodal Dataset Details

The same methods appear in [17].

#### Participants

Twenty-four (12 female) healthy, right-handed individuals between the ages of 19 and 35 (mean age: 23.08±3.87 years) with normal to corrected-vision were recruited for participation. All participants were screened and cleared for any neurological, psychiatric, substance abuse-related problems. All participants provided written informed consent in accordance with a joint Baycrest-University of Toronto Research Ethics Board Committee, which approved this study.

#### fMRI acquisition and analysis parameters

Participants were scanned in a Siemens Magnetrom TIM Trio Whole Body 3T MR scanner with a matrix 12-channel head coil. A structural MRI was obtained for each participant at the beginning of each scanning session, consisting of a 3D T1-weighted pulse sequence [echo time (TE), 2.6; 256×256 acquisition matrix, voxel size, 1.0×1.0×1.0 mm]. For functional scans, 28 oblique axial slices with full brain coverage were obtained [TR, 2 s; 64×64 acquisition matrix; voxel size, 3.125 mm×3.125 mm×5.0 mm] using T2*-weighted echo-planar image (EPI) sequence. Oblique axial slices were acquired to minimize sinus-related artifacts that occur in EPI sequences. These oblique axial slices were restored to the normal axial plane during reconstruction procedures.

Preprocessing of images was completed in Statistical Parametric Mapping (SPM5; available at http://www.fil.ion.ucl.ac.uk/spm/software/spm5/) and MELODIC [48] software packages. The images were corrected for slice timing differences in the EPI interleave acquisition, co-registered to the first EPI image in each run, corrected for gradient and residual head-motion artifacts using independent components analysis with MELODIC, spatially normalized to a standard MNI template with an affine transformation and 4d-spline interpolated and smoothed with an 8-mm Gaussian kernel. Functional data were parceled into 229 different ROIs based on sampling every 100th voxel using the AAL template as a gray matter mask.

#### Task Parameters

The experiment contained two types of auditory stimuli that were matched in amplitude but differed in frequency (250 Hz and 4000 Hz). The visual stimuli used in the experiment were a black and white checkerboard pattern (square) and the same checkerboard rotated 45 degrees (diamond). All stimulus presentations were controlled by Presentation software (version 10.2, Neurobehavioural Systems Inc.).

A trial was composed of a first stimulus (S1), delay, second stimulus (S2), response window and a variable inter-trial interval of 3, 5, 7, or 9 seconds. Two main task types were designed by manipulating the content of S1 and S2. For Cue 1st (C1) tasks, S1 was a cue that signaled a response compatibility rule to a subsequent lateralized target (S2). In Target 1st (C2) tasks, S1 was the lateralized target followed by the cue stimulus (S2). In a compatible trial, a cue instructed participants to press a button on the same side (left or right) as the lateralized target. In an incompatible trial, participant’s pressed the button on the opposite side of the lateralized target.

Auditory and visual stimuli were presented in Cue 1st and Target 1st tasks. In auditory cue/visual target sub-tasks, the pitch of a binaural tone specified the response rule to a lateralized visual target, a square checkerboard. Low (250 Hz) and high (4000 Hz) pitch tones signaled compatible and incompatible responses, respectively. In the visual cue/auditory target sub-tasks, the shape of a visual stimulus indicated the response rule to a lateralized, monaural tone of 250 Hz. Square and diamond checkerboards corresponded to compatible and incompatible responses. In summary, modality and stimulus presentation order manipulations resulted in four tasks: auditory cue followed by visual target (C1A), visual cue followed by auditory target (C1V), visual target followed by auditory cue (C2A) and auditory target followed by visual cue (C2V).

We used a mixed event-related experimental design for collecting fMRI data. Each of the four tasks were repeated three times for a total of twelve runs (9 mins, 28 seconds in duration) with 40 trials (20 compatible, 20 incompatible) randomly presented within each run. We collected data on two scanning days (6 runs per day) and counterbalanced across task types.

### MEG Dataset Details

The same methods appear in [18].

#### Participants

Fourteen healthy, right-handed (8 female) participants between the ages of 19 and 30 (mean age: 22±2 years) with normal or corrected-vision, were recruited for the study. All participants were screened and cleared for any neurological, psychiatric, substance abuse-related problems. All participants provided written informed consent in accordance with a joint Baycrest-University of Toronto Research Ethics Board Committee, which approved this study.

#### MEG acquisition and analysis parameters

MEG recordings were performed at the Rotman Research Institute at Baycrest Centre, in a dimly-lit, magnetically shielded room using a 151-channel whole-head axial gradiometer system (VSM-Med Tech Inc., Coquitlam, BC, Canada) with receiver coils uniformly spaced approximately 3 cm apart on a helmet-shaped array. Two separate blocks of MEG recordings took place, each approximately 18 minutes in length at a sampling rate of 625 Hz. The position of the participant’s head in the MEG was recorded at the beginning and the end of each data session using indicator coils placed on the nasion and bilateral preauricular points. Motion tolerance was set to 0.5 cm and all participants were within this limit. MEG data was acquired with participants lying in a supine position to minimize head movements while viewing a screen above the MEG system adjusted to participants’ eye level. Independent components analysis [ICA; [49]] was used to correct for ocular and cardiac artifacts (http://sccn.ucsd.edu/eeglab/). MEG data were parsed into 150 trials per task over the range −2.0 to 1.24 s relative to scene onset, band-passed between 0.5 and 55 Hz using a bidirectional filter, and time series were downsampled by a factor of four from the original digitization interval. The baseline interval chosen for correction was −0.5 to −1.0 s. The data were analyzed in a third-order gradient configuration for external noise correction. In addition to MEG recordings, a structural magnetic resonance image (MRI) was also acquired for each participant for co-registration with the MEG functional data. The structural image was a 3D MPRAGE T_1_-weighted pulse sequence (echo time, 2.6 ms; repetition time, 2 s; 256×256 acquisition matrix; voxel size, 1.0×1.0×1.0 mm) acquired on a 3 Tesla magnet (Siemens Magnetrom TIM Trio Whole Body scanner) located at Baycrest Centre.

A data-driven approach, previously applied to electroencephalographic data [50] was employed to derive brain sources/networks that gave rise to MEG signals recorded on the scalp. Preprocessed data were concatenated into a single matrix with MEG sensors (150) on the x-axis and participants (14), conditions (2), trials (150), and time samples (407) on the y-axis. The grand data matrix was evaluated with Bayesian Information Criterion [BIC; [51]] to determine the optimal subspace that fairly expressed the complexity of the data. The complete theoretical range of models (1–150) based on the rank of the smallest dimension of the data matrix were assessed. The probability of each model was estimated by leveraging goodness of fit and model complexity. The peak probability of the distribution for all models occurred at 34. Next, Principal Component Analysis (PCA) was performed on the original data matrix with the number of principal components (PCs) restricted to 34. Subsequently, ICA replaced the orthogonal PCs with independent components (ICs) that were not necessarily uncorrelated but maximally independent in the temporal domain. 2 out of the 34 components were dropped because they contained residual artifacts. The remaining 32 group ICs were projected onto participant data by multiplying the original time series for each trial/condition with the weighting (mixing) matrix. In this way, participant-specific ICs, representing sources/networks, were calculated with weights from group analysis.

The resulting spatial maps were translated into brain locations using an event-related vector beamformer [52], [53]. All 32 components showed dipolar patterns and were successfully mapped to mainly singular sources in MEG data.

For each individual, the functional maps obtained from source reconstruction were warped into a common space - the Montreal Neurological Institute (MNI) standard brain, using SPM software’s (http://www.fil.ion.ucl.ac.uk/spm/software/) spatial normalization engine. To infer group-level source distributions, normalized functional images were averaged for all participants. A peak coordinate from within an activated cluster was identified from group-averaged data. Using this procedure, a spatial location was assigned to each of the 32 ICs. Functional connectivity was estimated by correlating the beamformer-reconstructed activity for all pairs of sources.

#### Task Parameters

Visual stimuli were eight scenes (4 indoor, 4 outdoor) taken from a repository of images used in [54]. The experiment contained two tasks: active learning and choice reaction time with no learning component, with 150 trials per task. For learning, participants linked 2 scene pairs with 2 color pairs (4 associations) through trial-and-error. The general structure of a trial consisted of scene presentation (500 ms), a delay interval (750 ms) and a color pair, after which the participants had up to 1.2 sec to record their responses. Participants received on-screen, written feedback (correct/wrong) about their response choice. After responses were made, a jittered, random, inter-trial interval between 4.5 to 6 sec ensued. In the control task, participants were instructed in advance about which colors were correct and scenes preceding these colors were shown randomly. All stimuli were unique for both tasks and were randomized for individuals. The order in which the tasks were administered was counterbalanced across participants.

### PET Dataset Details

Information about the PET data set was compiled from a previously-published study by Cabeza, McIntosh, Tulving, Nyberg and Grady (1997). In that study, the authors constructed a structural equation model (SEM, also known as path analysis) to investigate how the patterns of interregional influence changed during memory encoding and memory recall, and whether these memory-related effects were modulated by aging. Specifically, data were taken from Tables 1 and 2, which show the coordinates of the nodes and the SEM-estimated beta coefficients (described in more detail below), allowing us to fully reconstruct the network for both the Encoding and Recall conditions.

The original PET data, which measured regional cerebral blood flow (rCBF), was processed in a manner analogous to “standard” BOLD signal processing, including motion correction, spatial normalization and spatial smoothing. The regional nodes of the network (n = 13) were selected from an initial exploratory analysis of the mean signal, i.e. all nodes showed a statistically significant main effect of memory task.

The data from Cabeza et al. (1997) study are unique in the sense that they represent a measure of effective connectivity, rather than functional connectivity. Thus, the connections express the direct effect one brain region has on another, above and beyond any effects of indirect connections. The values for the individual connections are the standardized path coefficients from the SEM analysis, which are analogous to regression (“beta”) coefficients and represent partial correlations (McIntosh & Gonzalez-Lima, 1994).

Specifically, the path coefficients were estimated as follows. An empirical matrix of regional covariances was calculated and an initial structural network was constructed using prior empirical knowledge about the anatomical connectivity among the 13 nodes of the network. The weight of each connection or path was assigned an initial value. Through an iterative data fitting procedure, the weights were modified to find an optimal solution, which best reproduced the empirically-observed pattern of regional covariances.

Details regarding the participants, task, acquisition parameters and preprocessing parameters can be found in [31]. When analyzing data from this published report, we examined differences in effective connectivity for older adults compared to younger adults in the recall condition. Euclidean distances between brain areas was calculated using the coordinates listed in Table 1 of that publication, while the path coefficients were compiled from Table 2 of that publication.

## Supporting Information

File S1 **Fisher-transformed correlation matrices.** Each of the Studies is represented as a MATLAB (The Mathworks, Natick, MA) cell array. Each cell represents an experimental group, and each array is organized in the following way: Conditions × Participants × Regions × Regions.
(MAT)
